# ﻿*Impatiensmaolanensis* (Balsaminaceae), a new species of *Impatiens* in a tiankeng from Guizhou, China

**DOI:** 10.3897/phytokeys.254.142981

**Published:** 2025-04-01

**Authors:** Bai-Zhu Li, Qin-Ying Wen, Jin-Dong Wang, Xiao-Xiang Huang, Zhi-Bin Xiong, Zhi-Juan Deng, Yin Yi, Xiao-Xin Tang

**Affiliations:** 1 School of Life Sciences, Central China Normal University, Wuhan 430079, China; 2 Key Laboratory of National Forestry and Grassland Administration on Biodiversity Conservation in Karst Mountainous Areas of Southwestern China, School of Life Science, Guizhou Normal University, Guiyang 550025, China; 3 Key Laboratory of Plant Physiology and Developmental Regulation, School of Life Science, Guizhou Normal University, Guiyang 550025, China; 4 Maolan National Nature Reserve Administration Bureau, Qiannan Buyei and Miao Autonomous Prefecture 558400, China

**Keywords:** Balsaminaceae, morphology, new species, SEM, taxonomy

## Abstract

*Impatiensmaolanensis* Z.B.Xiong & Q.Y.Wen (Balsaminaceae), a new species of Impatienssubg.Clavicarpa from Maolan National Nature Reserve, Guizhou, China, is described. The new species grows in a tiankeng (a large, naturally formed pit) connected to a dried-up underground river. *I.maolanensis* is similar to *I.auriculata* Chang Y. Xia & S. X. Yu, *I.liboensis* K. M. Liu & R. P. Kuang and *I.tianlinensis* S. X. Yu & L. J. Zhang, but differs from the latter three species in terms of orange-red flowers, roots, stems, bracts, dorsal petals, lateral sepals, lower sepals, pollen grains and seeds micromorphology. The micromorphological characteristics and surface patterning of pollen grains and seeds of the new species were examined using scanning electron microscopy (SEM). Pollen grains of *I.maolanensis* are triangular-round in polar view and elliptical in equatorial view. The pollen exine has an irregular and relatively smooth reticulate ornamentation, and under high magnification, granular protrusions can be observed. Seeds of *I.maolanensis* are black and narrowly ellipsoid. The seed coat has reticulate ornamentation with slightly sunken meshes, folded base, and granular protrusions within the meshes. Morphological and micromorphology evidence support the establishment of the new species. Our study provides detailed information on the new species, including morphological characteristics, phenology, photographs, palynology, seed micromorphology, etymology, habitat and distribution, and conservation assessment.

## ﻿Introduction

The genus, *Impatiens* L., belonging to the family Balsaminaceae, is a large genus of angiosperms with approximately 1,000 species around the world (Grey-Wilson 1980; Janssens et al. 2006; Yu et al. 2016). It comprises two subgenera, subg. Clavicarpa S. X. Yu ex S. X. Yu & Wei Wang and subg. Impatiens (Yu 2012; Yu et al. 2016; Zeng et al. 2016). *Impatiens* is widely distributed in the Northern Hemisphere, primarily in tropical and subtropical regions (Yu 2012). Most species within this genus are herbaceous plants, characterized by distinct floral structures consisting of one or two pairs of lateral sepals, a dorsal petal, two lateral united petals, and a lower sepal with a nectar spur (Yu et al. 2016). The morphological variation within *Impatiens* is exceedingly intricate, presenting numerous challenges to plant taxonomists (Chen 2001; Ruchisansakun et al. 2015; Li et al. 2022).

In China, more than 270 species of *Impatiens* are discovered, distributed across the country, with a concentration primarily in the southwest. Among them, more than 240 species are endemic to China, and the majority are narrow endemics, particularly in karst areas such as Yunnan, Guizhou, Sichuan, and Guangxi, where the endemism phenomenon is particularly pronounced (Yu 2012). Over recent years, a plethora of new species has been successively discovered in the areas (Yuan et al. 2011; Kuang et al. 2014; Cai et al. 2015; Luo et al. 2015; Tan et al. 2015; Ding et al. 2016; Cho et al. 2017; Xia et al. 2019; Qin et al. 2020; Gu et al. 2021; Liao et al. 2021, Song et al. 2021a, b; Yuan et al. 2022; Wang et al. 2022a, 2022b; Yuan et al. 2022; Zhang et al. 2023a; Hu et al. 2024; Song et al. 2024). Thus, the plants of *Impatiens* can be characterized by the phrase “one species per mountain, one species per cave, one species per karst basin” (where “karst basin” denotes a small basin encircled by multiple limestone hills within a karst landscape) (Yu 2012). Traditionally, morphological characteristics, pollen grains and seed micromorphology have been considered essential factors for distinguishing species and classifying them within *Impatiens* (Lu and Chen 1991; Yu 2012; Zeng et al. 2016).

In October 2024, during a field survey conducted at the Maolan National Nature Reserve, Libo County, Qiannan Buyei and Miao Autonomous Prefecture, Guizhou, China, we collected a species of *Impatiens* in a tiankeng connected to a dried-up underground river. According to Yu Shengxiang’s classification, this species belongs to the subgenus Clavicarpa S. X. Yu ex S. X. Yu & Wei Wang of *Impatiens* in family Balsaminaceae (Yu 2012; Yu et al. 2016). Plants belonging to this subgenus are generally perennial herbs, distinguished by racemes with more than 5 flowers, 4 lateral sepals, 4-loculed carpels with a single seed per locule, clavated fruits, elliptical seeds, and 3-colpated pollen grains as key characteristics (Yu 2012). *Impatiensmaolanensis* is similar to *I.auriculata* Chang Y. Xia & S. X. Yu, *I.liboensis* K. M. Liu & R. P. Kuang and *I.tianlinensis* S. X. Yu & L. J. Zhang in terms of morphology, floral structure, and capsule shape (Yu 2012; Kuang et al. 2014; Zeng et al. 2015; Zeng et al. 2016). However, it exhibits significant differences from the latter three species in terms of orange-red flowers, roots, stems, bracts, dorsal petals, lateral sepals, lower sepals, pollen grains, and seeds micromorphology.

Therefore, in early November 2024, we conducted a survey in Guizhou to collect flowering materials of the species. After careful observation of morphological characteristics, pollen grains and seed morphology, and following a comprehensive comparison with known *Impatiens* species (*I.auriculata*, *I.liboensis* and *I.tianlinensis*) (Chen 2001; Yu 2012; Kuang et al. 2014; Zhang et al. 2014; Zeng et al. 2015; Zeng et al. 2016; Zhang et al. 2023b) we confirmed this species as a new species and provide its description below.

## ﻿Material and methods

### ﻿Morphological and micromorphological analysis

This study integrates data from herbarium specimens, digitized specimen images, field observations, and taxonomic literature. Specimens were meticulously examined through visits to the Institute of Botany, Chinese Academy of Sciences (**PE**), Guangxi Institute of Botany (**IBK**), Guangxi Medicinal Botanical Garden (**GXMG**), and Hunan Normal University (**HNNU**) (herbarium acronyms follow *Index Herbariorum*; Thiers, 2025). Digital images were additionally sourced from the Chinese Virtual Herbarium (**CVH**; https://www.cvh.ac.cn/). The taxonomic description followed the terminology used by Chen (2001) and Yu (2012). The holotype voucher specimens were stored at the Herbarium of Guizhou Normal University (**GZNU**). The conservation status of the new species was assessed following the guidelines of the IUCN Red List Categories and Criteria (IUCN 2024).

At the same time, we collected plant specimens, mature pollen grains, and seeds of *I.maolanensis*, *I.auriculata* and *I.liboensis* from Maolan National Nature Reserve, Libo County, Qiannan Buyei and Miao Autonomous Prefecture, Guizhou, China. We measured the plant height with a tape measure and used a vernier caliper to measure the flower characteristics of fresh plants. The collected mature pollen grains and seeds were enveloped in absorbent paper and subsequently placed into paper bags with silica gel for drying. Dried pollen grains and seeds were carefully attached to stubs with the aid of double-sided adhesive tape and then thinly coated with gold, approximately 2 nm in thickness, using a MSP-1S sputter coater for a duration of 90 seconds. The pollen grains and seeds coated with gold were subsequently observed and photographed using a HITACHI-SU8600 scanning electron microscope. The polar axis and equatorial axis diameters of 30 pollen grains, as well as the length and width of the seeds, were measured respectively. Micromorphological characteristics of the pollen grains and seeds were described following the methods outlined by Walker and Doyle (1975), Wang and Wang (1983), and Lu and Chen (1991). The morphological and pollen grains’ micromorphological comparison was conducted between *I.maolanensis* and *I.tianlinensis*; the latter species was described in detail by Zeng et al (2015, 2016).

## ﻿Results

### ﻿Taxonomic treatment

#### ﻿ Impatiens maolanensis

Taxon classificationPlantaeEricalesBalsaminaceae

Zhibin Xiong & Qinying Wen sp. nov.

3812F32D-A140-5AA3-8DD8-30AA50F2E5B8

urn:lsid:ipni.org:names:77359625-1

**Figure 1.**
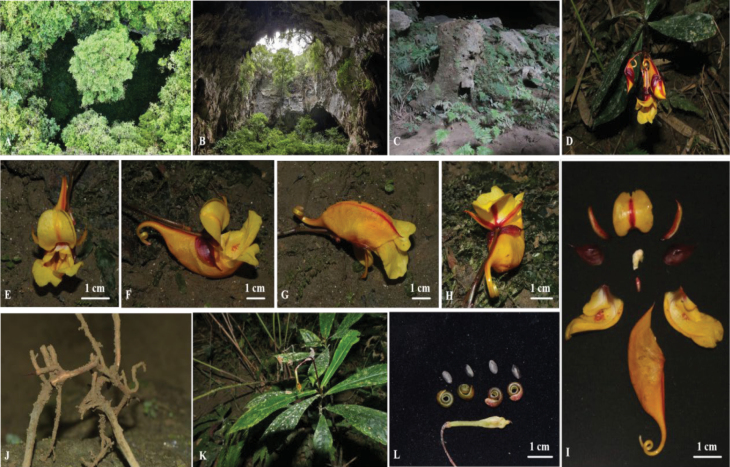
Habitat and morphology of *Impatiensmaolanensis* Zhi-Bin Xiong & Q.Y.Wen, sp. nov. **A–C** habitat **D** plant **E** front view of flower **F–H** different views of flower **I** anatomy of flower **J** root **K** capsule **L** seed (Photographed by Zhi-Bin Xiong and Qin-Ying Wen).

Figs 1 , 3A–D , 4A–C

##### Diagnosis.

**Figure 2.**
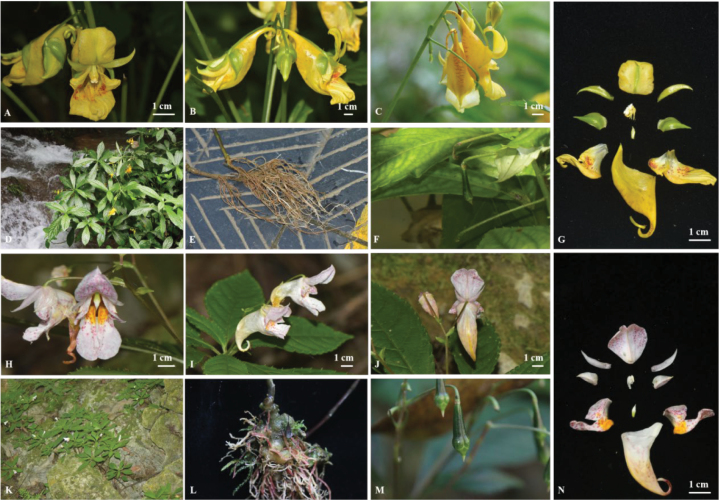
*Impatiensauriculata***A** front view of flower **B, C** different views of flower **D** habitat **E** root **F** capsule **G** anatomy of flower. *Impatiensliboensis***H** front view of flower **I, J** different views of flower **K** habitat **L** root **M** capsule **N** anatomy of flower (Photographed by Qin-Ying Wen and Bai-Zhu Li).

**Figure 3.**
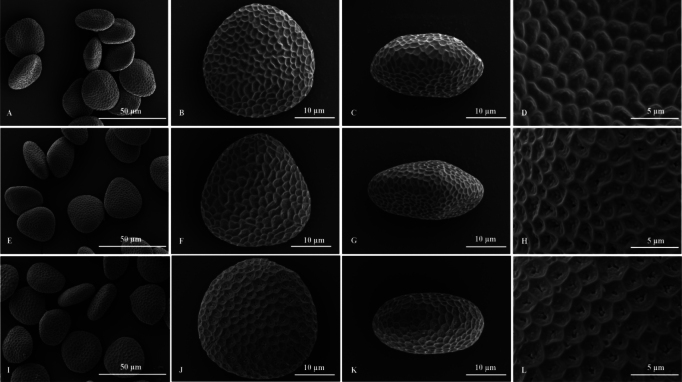
Scanning electron micrographs of pollen grains. *Impatiensmaolanensis* Zhi-Bin Xiong & Q.Y.Wen, sp. nov. **A** group view, ×1000 **B** polar view, ×3000 **C** equatorial view, ×3000 **D** exine ornamentation, ×7000. *Impatiensauriculata***E** group view, ×1000 **F** polar view, ×3000 **G** equatorial view, ×3000 **H** exine ornamentation, ×7000. *Impatiensliboensis***I** group view, ×1000 **J** polar view, ×3000 **K** equatorial view, ×3000 **L** exine ornamentation, ×7000 (Photographed by Bai-Zhu Li).

**Figure 4.**
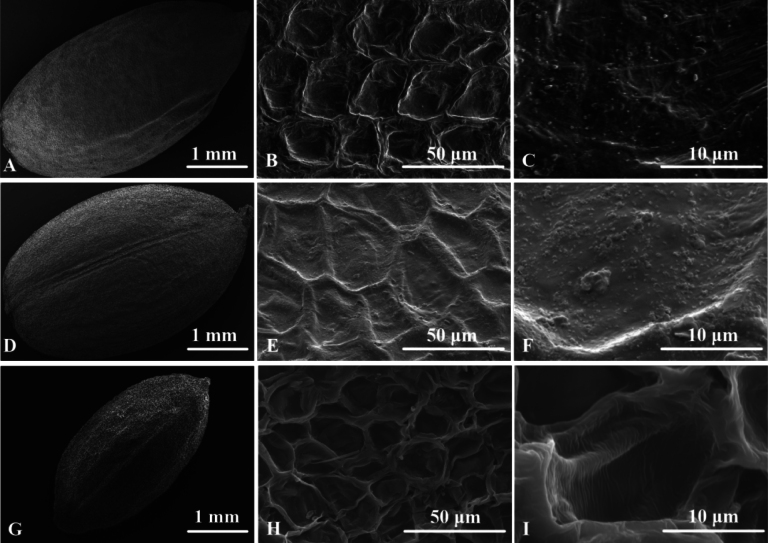
Scanning electron micrographs of seeds. *Impatiensmaolanensis* Zhi-Bin Xiong & Q.Y.Wen, sp. nov. **A** whole view, ×30 **B** partial view, ×1000 **C** partial view, ×5000. *Impatiensauriculata***D** whole view, ×30 **E** partial view, ×1000 **F** partial view, ×5000. *Impatiensliboensis***G** whole view, ×30 **H** partial view, ×1000 **I** partial view, ×5000 (Photographed by Bai-Zhu Li).

*Impatiensmaolanensis* is similar to *I.auriculata* (Figs 2A–G, 3E–H, 4D–F), *I.liboensis* (Figs 2H–N, 3I–L, 4G–I) and *I.tianlinensis* (Zeng et al. 2015, 2016) but its characteristics are significantly different from the latter three species in terms of orange-red flowers, thick fibrous root, stem with leaf scars and nodes, petioles, sessile or nearly sessile, bracts, outer lateral sepals, dorsal petal, lower sepal, and fruit color (Table 1).

**Table 1.** Detailed comparison of *I.maolanensis*, *I.auriculata*, *I.liboensis* and *I.tianlinensis*.

Characters	*I.maolanensis*	*I.auriculata*	*I.liboensis*	*I.tianlinensis*
Habitat	perennial	annual	perennial	perennial
Plant height	25–35 cm	50–160 cm	22–50 cm	50–80 cm
Root	thick fibrous root	fibrous root	globose or irregular underground tuber	-
Stem	erect, branched, with leaf scars and nodes	erect, branched	erect, unbranched	fleshy, erect, simple, robust; inferior nodes swollen
Leaves	over-oblong or ovate-lanceolate	oblong-ovate	ovate-oblong or nearly oblong	obovate to oblanceolate
petiole	sessile or nearly sessile	sessile or nearly sessile	1.5–5 cm long	(0.5-) 1–2 cm long (or upper leaves subsessile) with several short clavate glands
Flower color	orange-red and vertical stripes	yellow-red spots and markings	white or pink white	yellowish or cream
Inflorescence	4-flowered	3–11 flowered	3–7 flowered	3–5 (-7) flowered
Bracts	ovate with long-cuspidate apex, red	ovate or oblong-ovate, green	ovate or oblong-ovate, green	ovate, acute, deciduous
Outer lateral sepals	obliquely ovate, dark red	obliquely ovate, green	obliquely ovate, white-pink	ovate, symmetric, yellowish-green
Inner lateral sepals	linear-lanceolate, orange adaxial surface, orange with a red stripe on abaxial surface,	linear-lanceolate, yellow-green	linear-lanceolate, white-pink	sickle-shaped, inaequilateral, apex acuminate or caudate
Dorsal petals	oblong, orange, cuneate at base, obtuse and emarginate at apex, with abaxial midvein thickened and a vertical red stripe on both adaxial and abaxial surface	obovate or nearly round, yellow-green, obtuse at apex, emarginate, with abaxial midvien thickened	obovate, white-pink, round at base, obtuse at apex, emarginate, with abaxial midvien thickened, obviously but narrowly carinate	ovate, apex obtuse, base broadly cuneate, midrib obvious, with a slight dorsal crest
Lateral united petals	basal lobes, orange, nearly oblong, obtuse at apex; disal lobes, orange-red spots, obovate-oblong, obtuse at apex and near middle retuse, with abaxial auricle inflexed, suborbicular	basal lobes, yellow-red spots, nearly oblong, obtuse at apex; disal lobes, yellow-red spots, obovate-oblong, obtuse at apex, with with abaxial auricle inflexed, semi-ovate	basal lobes, white-yellow and red spots, nearly oblong, acute at apex; disal lobes, white-yellow and red spots, obovate-oblong or obliquely obvate, obtuse at apex and near middle retuse,with abaxial auricle inflexed, suborbicular	basal lobes, yellow-red spots, oblong; disal lobes, yellow-red spots, elliptic, yellow-apex emarginate, middle of inner margin without appendage
Lower sepal and spur	saccate, orange-red, with an incurved or spiraled spur, 3–4 cm long, with a vertical red stripe on the top and base of surface	funnel, yellow-red spots and markings, with a long inwardly curved spur	saccate, white-pink spots, with an incurved spur, 1.2–1.5 cm long	saccate, abruptly constricted into an involute spur, 1–1.5 cm long
Capsule	obovate-clavate, drak green-dark red, with a red beak at apex	obovate-clavate, green, with a beak at apex	obovate-clavate, dark green, swollen in the upper part rostellate at apex	hammer-shaped
flowering season	September to November	October to December	August to November	September to November
fruiting season	October to December	November to January	September to November	September to November
Pollen grains	triangular-round in polar view, three-colpate, exine has an irregular reticulate ornamentation	nearly triangular in polar view, three-colpate, exine has an irregular reticulate ornamentation	nearly triangular in polar view, three-colpate, exine has an irregular reticulate ornamentation	triangular in polar view, exine has an irregular reticulate ornamentation, and there are hardly any granular protrusions within the mesh under high magnification
Seeds	narrowly ellipsoid, 4.5 mm long, 2.42 mm wide, black, reticulate ornamentation with slightly sunken meshes, folded base, and granular protrusions within the meshes	ellipsoid, 4.22 mm long, 2.71 mm wide, brown, reticulate ornamentation with slightly sunken meshes, folded base, and granular protrusions within the meshes	ellipsoid, 3.48 mm long, 1.76 mm wide, brown, reticulate ornamentation with sunken meshes, folded base, and no granular protrusions within the meshes	ellipsoid

##### Type.

China • Guizhou Province, Qiannan Buyei and Miao Autonomous Prefecture (黔南布依族苗族自治州), Libo County (荔波县), Maolan National Nature Reserve (茂兰国家级自然保护区), Karst terrain, 25°19'59"N, 108°2'58"E, alt. 541 m, 28 October 2024, *Zhibin Xiong and Qinying Wen* (holotype: GZNU2024102801!, isotypes: GZNU2024102802!, GZNU2024102803!).

##### Etymology.

The specific epithet ‘maolanensis’ refers to the locality where this new species was discovered, located in Maolan National Nature Reserve, Libo County, Guizhou Province, China. The new species is named ‘茂兰凤仙花’ in Chinese.

##### Description.

Plants perennial, 25–35 cm tall. Roots fibrous, 0.5 cm thick or thicker, up to 9 cm long. Stem robust, erect, branched, 0.4 cm thick or thicker, with leaf scars and nodes. Leaves alternate, densely arranged at the top of stem, glabrous, deep green, membranous, 9–12.5 cm long, 2.5–4 cm wide; petiole, sessile or nearly sessile, with two glands at base; lamina 10.4–13.6 cm long, 3.1–3.7 cm wide, over-oblong or ovate-lanceolate, acuminate to cuspidate at apex, cuneate at base, with crenate margin, setose between marginal teeth; lateral veins in 6–9 pairs. Inflorescences in upper leaf axils, racemose, 2–4 flowered, peduncle 5–7 cm long, dark green to dark red; pedicels 1–2 cm long, dark red; bract 1, at base or middle of pedicel, persistent, bracts ovate with long-cuspidate apex, 3–5 mm long, dark red. Flowers orange-red, 4–5 cm long. Lateral sepals 4; outer lateral sepals 2, obliquely ovate, 1.5 cm long, 0.7 cm wide, dark red, acute at apex, with abaxial midvein slightly thickened; inner lateral sepals 2, linear-lanceolate, 1.5 cm long, 0.28 cm wide, orange adaxial surface, orange with a red stripe on abaxial surface, recurved at apex. Lower sepal saccate, gradually elongates into an incurved or spiraled spur, 3–4 cm long, with a vertical red stripe on the top and base of surface, 4 cm long (excluding spur); mouth obliquely upwards, 1.2–1.5 cm wide, acute at apex. Dorsal petal oblong, 1.2 cm in diameter, orange, cuneate at base, obtuse and emarginate at apex, with abaxial midvein thickened and a vertical red stripe on both adaxial and abaxial surface. Lateral united petals sessile, orange with red spots, 2-lobed, 2.3–2.6 cm long; basal lobes, orange, nearly oblong, 1.1 cm long, 0.5 cm wide, obtuse at apex; disal lobes, orange-red spots, obovate-oblong, 2.1 cm long, 0.9 cm wide, obtuse at apex and near middle retuse, with abaxial auricle inflexed, suborbicular. Stamens 5, ca. 5 mm long; filaments linear; anther small, ovate, obtuse at apex. Ovary superior, 4-carpellate, erect with axile placentation, fusiform, red, ca. 5 mm long; stigma four-lobed, red. Capsule obovate-clavate, with a red beak at apex, deep green to dark red, 1.5–2 cm long, 4-valved, fleshy. Seeds narrowly elliptical, 4.5 mm long, 2.42 mm wide, black.

##### Phenology.

Flowering season: September to November. Fruiting season: October to December.

##### Palynology.

Pollen grains of *Impatiensmaolanensis*, *I.auriculata*, *I.liboensis* and *I.tianlinensis* are three-colpate, with an exine with irregular reticulate ornamentation. Pollen grains of *I.maolanensis* are triangular-round in polar view and elliptical in equatorial view, with a polar: equatorial ratio of 28.4–32.5: 28.2–32.1 μm. The pollen exine has an irregular and relatively smooth reticulate ornamentation, and under high magnification, granular protrusions can be observed (Fig. 3A–D). Pollen grains of *I.auriculata* are nearly triangular in polar view and elliptical in equatorial view, with a polar: equatorial ratio of 27.5–32: 26.3–31.2 μm. The pollen exine has an irregular reticulate ornamentation and holes (Fig. 3E–H). Pollen grains of *I.liboensis*, are subellipsoid in polar view and elliptical in equatorial view, with a polar: equatorial ratio of 29.6–32.8: 28.7–32.6 μm. The pollen exine has an irregular reticulate ornamentation and holes (Fig. 3I–L). Pollen grains of *I.tianlinensis* are triangular in polar, with a polar: equatorial ratio of 29.62–30.47: 12.68–13.54 μm. The pollen exine has an irregular reticulate ornamentation and holes, and there are hardly any granular protrusions within the mesh under high magnification (Zeng et al. 2015, 2016).

##### Seed micromorphology.

Seeds of *Impatiensmaolanensis*, narrowly ellipsoid, 4.5 mm long, 2.42 mm wide, black. The seed coat has reticulate ornamentation with slightly sunken meshes, folded base, and granular protrusions within the meshes (Fig. 4A–C). Seeds of *I.auriculata*, ellipsoid, 4.22 mm long, 2.71 mm wide, brown. The seed coat has reticulate ornamentation with slightly sunken meshes, folded base, and granular protrusions within the meshes (Fig. 4D–F). Seeds of *I.liboensis*, ellipsoid, 3.48 mm long, 1.76 mm wide, brown. The seed coat has reticulate ornamentation with sunken meshes, folded base, and no granular protrusions within the meshes (Fig. 4 G–I).

##### Habitat and distribution.

**Figure 5.**
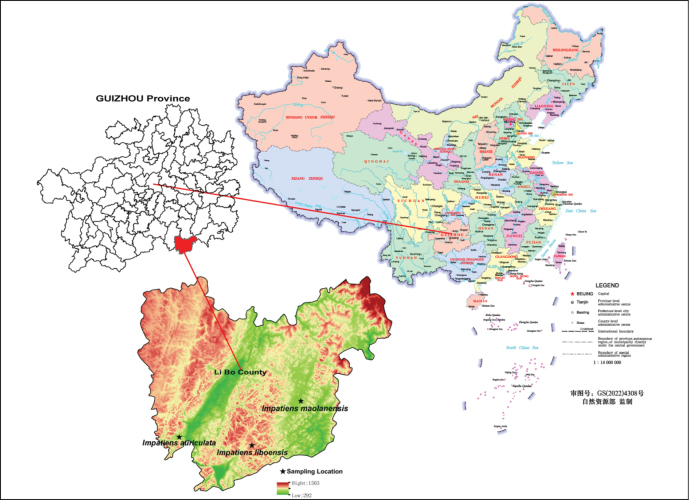
Map shows the sampling location of the three *Impatiens* species (Drawn by Jin-Dong Wang).

*Impatiensmaolanensis* has only been observed within the Maolan National Nature Reserve, Libo County, Qiannan Buyei and Miao Autonomous Prefecture, Guizhou Province, China (Figs 1A–C, 5). Its uniqueness lies in its growth within a tiankeng (a large, naturally formed pit or depression in the earth’s surface) at an altitude of 541 m, within the typical karst area. To reach this location, one must first pass through a karst cave, descend approximately 100 m to a dried-up underground river, and then continue for about 200 m to reach the tiankeng where the species grows. In terms of ecological coexistence, *I.maolanensis* grows alongside various plants such as *Sphagnum* sp., ferns, bamboo, *Pilea* sp., and *I.liboensis*.

##### Conservation status.

Our study documented a single population of 18 mature individuals within Guizhou Province, but no expanded surveys have been conducted in adjacent regions or potential habitats (e.g., karst landscapes in Guangxi or Yunnan). The conservation status of Impatiensmaolanensis is currently assessed as Data Deficient (DD) under IUCN Red List Categories and Criteria. While the observed population size suggests potential vulnerability, the lack of comprehensive geographic sampling prevents robust assessment against extinction risk criteria (e.g., CR D).

##### Similar species.

The new species, *I.maolanensis* is similar to *I.auriculata*, *I.liboensis* and *I.tianlinensis* in floral morphology. All species have alternate leaves, inflorescences, 4 lateral sepals, superior ovaries, 4 carpels, fusiform or obovate-clavate fruits, and three-colpate pollen grains. Nevertheless, the new species is easily distinguishable. Unlike the latter three species, *I.maolanensis* has a thick fibrous root; stem robust, with leaf scars and nodes; petioles, sessile or nearly sessile; flowers orange-red; bracts and outer lateral sepals, dark red; inner lateral sepals with a red stripe on abaxial surface; dorsal petal oblong, cuneate at base, obtuse and slightly emarginate at apex, with a red stripe on both the adaxial and abaxial surfaces; disal lobes, obovate-oblong, obtuse at apex and near middle retuse, orange with red spots; lower sepal saccate, gradually elongates into an incurved or spiraled spur, mouth obliquely upwards, and a red stripe on the top and base of surface; stigma red; capsule obovate-clavate, with a red beak at apex. The plant height of *I.maolanensis* is similar to *I.liboensis*, but significantly lower than *I.auriculata* and *I.tianlinensis*. The pollen grains’ micromorphology of *I.maolanensis* differ from the latter three species in that the pollen grains are triangular-round in polar view. The pollen exine has an irregular and relatively smooth reticulate ornamentation, and the meshes are almost without holes. The seeds’ micromorphology of *I.maolanensis* differs from *I.liboensis* and *I.auriculata* in that the seeds are black and narrowly ellipsoid. The seed coat has reticulate ornamentation with slightly sunken meshes, folded base, and granular protrusions within the meshes. *I.maolanensis*, *I.liboensis* and *I.auriculata* can be found within the Maolan National Nature Reserve in Libo County (Fig. 5). However, *I.maolanensis* is only discovered growing in a semi-shaded tiankeng, while *I.auriculata* grows near water, and *I.liboensis* grows under forests in shady and damp places or beside ditches. More detailed comparison of the four species is presented in Table 1.

## ﻿Discussion

Based on morphological characteristics and molecular evidence, Yu et al (2016) divided the genus Impatiensintosubgen.Clavicarpa and subgen. Impatiens. Our new species, *I.maolanensis* belongs in sect. Clavicarpa, subgen. Clavicarpa because of its 4-flowered, racemose inflorescence, 4 lateral sepals, lower sepal saccate or funnel, 4-carpellate ovary, obovate-clavate capsule, three-colpate pollen grains. The endemic species of *Impatiens* in limestone areas such as Yunnan, Guangxi, and Guizhou are the most abundant, and they are strongly differentiated by edge effects and small habitat changes. Some endemic species of limestone areas have grown into perennial fruticeous-herbaceous plants over a long evolutionary process, which is particularly special for herbaceous *Impatiens*. *I.maolanensis* is exclusively found in the tiankeng of karst areas, and it exhibits very distinct differences from similar species. It is known to exist as a single population comprising only 18 mature individuals. Therefore, we propose the establishment of a specialized conservation area to ensure the survival and promote the propagation of *I.maolanensis*.

## Supplementary Material

XML Treatment for Impatiens maolanensis

## ﻿Additional information

### Author ORCIDs

Bai-Zhu Li https://orcid.org/0000-0002-7227-097X

Qin-Ying Wen https://orcid.org/0009-0007-0438-1073

Jin-Dong Wang https://orcid.org/0009-0005-8020-3266

Yin Yi https://orcid.org/0000-0003-3079-334X

Xiao-Xin Tang https://orcid.org/0000-0003-0569-0453

### Data availability

All of the data that support the findings of this study are available in the main text.
